# Beyond semantics: Prosody can shape and modulate listeners’ emotional experience of poetry — A cross-linguistic investigation

**DOI:** 10.1371/journal.pone.0345072

**Published:** 2026-09-08

**Authors:** Yan Li, Ziqing Huang, Yannan Zhang, Yufeng Chen

**Affiliations:** 1 College of Arts, Northeastern University, Shenyang, China; 2 Foreign Studies College, Northeastern University, Shenyang, China; 3 School of Literature, Shandong University, Jinan, China; The Education University of Hong Kong, HONG KONG

## Abstract

Expressive prosody plays an important role in conveying emotion during oral poetry performance. This study investigated whether emotional prosody can serve as a perceptually robust cue for emotion judgment when semantic comprehension is unavailable. Experiment 1 confirmed systematic acoustic differences between joyful and sad poem recitations: joyful recitations exhibited higher normalized pitch, greater vocal intensity, and faster articulation rate, whereas sad recitations exhibited lower normalized pitch, reduced intensity, slower articulation rate, and reduced harmonicity. Experiment 2 employed a 3 (prosody–semantics alignment: congruent, incongruent, neutral) × 2 (valence: joy vs. sadness) × 2 (listener group: native Mandarin vs. Mandarin-naive Japanese listeners) design. When prosody and semantics were aligned, both listener groups produced the strongest emotion ratings consistent with the poems’ intended valence. Critically, Mandarin-naive Japanese listeners, who had no access to Chinese semantic content, showed strong modulation of affective judgments by available prosodic cues when vocal expression conflicted with the poems’ textual valence. Native Mandarin listeners integrated semantic and expressive prosodic information, but prosodic manipulations substantially weakened semantic biases and, in some cases, shifted semantically driven judgments. These findings suggest that prosodic configuration is an important component of expressive vocal realization in poetic recitation, shaping listeners’ affective responses alongside semantic content. Arousal-related acoustic variation may further contribute to these prosodic effects.

## Introduction

Lyricism constitutes a fundamental essence of poetry and one of the key elements of its aesthetic value. As the renowned British poet Byron remarked, “Poetry should express emotion” [1]. The ancient Chinese literary theorist Lu Ji (3rd century CE) similarly observed that “poetry originates from emotion and is ornate in expression” (诗缘情而绮靡). Thus, even across diverse cultural and historical contexts, lyricism remains a core characteristic of poetry [2,3].

Psychologists have conducted both theoretical and empirical investigations into certain emotional effects and aesthetic experiences elicited by poetry. Johnson-Laird and Oatley (2022) recently proposed three simulation models to address the fundamental question of how poetry evokes specific emotions [4]. The authors argue that, first, the semantic content of poetry can trigger emotional resonance; second, mimetic simulations of audible cues elicit particular emotional experiences; and third, when readers engage in self-simulation while perceiving the poem, they become aware of their immersion, thereby generating aesthetic emotions.

Contemporary experimental research has illuminated the mechanisms of emotional arousal through poetic semantic content. Factors such as the vividness of imagery in the text [5] and word valence [6,7] have been shown to influence readers’ emotional experiences of poetry. Regarding the third simulation proposed by Johnson-Laird and Oatley (2022), relevant experimental studies indicate that poems expressing the moods of persons, situations, or objects engage readers in mentally simulating and affectively resonating with the depicted state of affairs. This resonance can lead to the experience of the depicted mood itself or a closely associated feeling—a process akin to empathy understood as a form of Einfühlung or “feeling into.” Specifically, familiarity and situational embedding were identified as the primary factors mediating mood empathy, whereas aesthetic liking was most strongly predicted by foregrounding features such as style and form [8]. Another study revealed that personality traits influence subjective experiences of poetry, with the aesthetic appeal of a poem moderated by openness, intellect, awe-proneness, and epistemic curiosity [9].

The aforementioned simulations or experiences typically require the recipient to possess knowledge of the poem’s compositional context or sufficient comprehension of its meaning in order to achieve the corresponding aesthetic response. Beyond textual content, however, the aesthetic experience and emotional resonance of poetry can also be shaped by its structural features (i.e., poetic form) [10]—that is, the second type of simulation emphasized by Johnson-Laird and Oatley (2022). This mechanism operates even across languages and cultures.

Across cultures worldwide, poetry has never been confined to the silent page or the purely visual experience of reading; it has frequently been performed as material for recitation or song, thereby engaging the auditory faculty. In ancient Greece, epic poetry was declaimed aloud by professional performers known as *rhapsōidos* (rhapsodists or reciters) during festivals or competitions. Lyric poetry, such as that of Sappho, was closer to song and was typically accompanied by the lyre. In the Chinese tradition, classical poetry has been transmitted as “sung poetry” (*ge shi*) since the era of the *Shijing* (Book of Odes); many of its pieces were originally composed for musical performance and singing.

The vocal performance of poetry has given rise to a fundamental hypothesis concerning phonetic iconicity: that particular linguistic sounds can, in themselves, symbolize or evoke specific sensations, images, or emotions. Consequently, scholars have sought to establish a verifiable, quantifiable correlation between the formal sonic structure of poetry and the emotional perceptions of listeners. Currently, three main theoretical perspectives have emerged in the field, which we refer to as the phoneme-frequency hypothesis, the emotional-rhyme hypothesis, and the emotional-prosody hypothesis, respectively.

The phoneme-frequency hypothesis posits a relationship between the frequency of vowels and consonants and the perception of emotion in poetry. Some studies have identified emotion-specific classificatory differences in poetic phoneme inventories by quantifying the occurrence of particular segments. As early as 1876, Gustav Theodor Fechner, a founder of empirical aesthetics, proposed that, in general, “a, e, i appear as brighter and o, u as darker.” [11] Subsequently, M. M. Macdermott (1940), through statistical analysis of English poems, observed that dark vowels predominate in lines referring to dark colors, mystical obscurity, slow and heavy movement, or depicting hatred and struggle [12]. However, vowels of every type appear in most poems and may therefore neutralize one another’s emotional evocations. More recent attempts to replicate emotional effects attributable to vowels have largely failed. Velichkova and Voropaeva (2023) conducted an experiment to test E. N. Vinarskaya’s hypothesis, which links joy or excitement to front vowels (e.g., /i/, /e/)—produced with a raised tongue front and facial musculature resembling a “smile”—while associating sadness or melancholy with slower, deeper breathing, sighing, relaxed throat muscles, and a larger or neutral oral aperture, favoring central and back vowels (e.g., /a/, /o/, /u/). Contrary to theoretical predictions, their analysis of “sad” emotions in a German poetry corpus revealed that even poems unanimously judged as sad were dominated by front and central vowels (I-, E-, and A-timbres) [13].

Other studies have examined the relationship between consonant frequency and emotional classification in poetry. FóNagy (1961) analyzed works by prominent poets (e.g., Hungary’s Petőfi, France’s Hugo and Verlaine, and Germany’s Rückert) and found that, in poems expressing “aggressive” emotions, plosives (particularly /k/ and /t/) and the trill /r/ occurred significantly more frequently than average [14]. In “tender” poems, by contrast, nasals (/m/, /n/) and the lateral approximant /l/ predominated. Later research [15,16], however, suggested that relatively high plosive frequency is more likely to characterize highly activated pleasant emotions, whereas high nasal frequency characterizes low-activation unpleasant emotions. These studies nevertheless exhibit clear selection bias: they focused on specific segments rather than complete phoneme classes (plosives and nasals in their entirety). Notably, their conclusions have also faced replication difficulties [17].

The emotional-rhyme hypothesis maintains that rhyme schemes and the selection of specific rhymes can themselves embody or convey distinct emotions. Within the simulation framework proposed by Johnson-Laird and Oatley (2022), adjacent rhymes and alliterations should convey a relatively upbeat tempo. In studies of Chinese poetry, numerous empirical observations have likewise linked emotional expression to rhyming characteristics. For instance, Zhe (2014) categorized Chinese rhyme finals into three groups—“sonorous,” “soft,” and “subtle”—corresponding respectively to expressions of joy, melancholy, and grief in Du Fu’s poetry [18]. Similarly, Shu (2002) argued that poems ending in the obsolete check tone (*rù shēng*) tend to express suppressed indignation or sorrow, owing to the abrupt and truncated quality of this phonetic category [19]. These impressionistic claims, however, have not received broad experimental or statistical support. Although Menninghaus et al. (2015) found that parallelistic features—including meter and rhyme—function as general intensifiers of emotional impact irrespective of whether the poem’s dominant emotion is sad or joyful, it must be emphasized that this effect pertains solely to the amplification of pre-existing emotional content rather than to the shaping of an emotional experience [20].

Recent scholarship has shifted attention to the influence of poetic prosody on emotional experience—an approach centered on what we refer to as the emotional-prosody hypothesis. Here, “prosody” is operationalized primarily from an acoustic-phonetic perspective, referring to surface suprasegmental features such as pitch, intensity, pauses, and lengthening, in line with conventional phonetic definitions. In this sense, our analysis focuses on holistic acoustic summaries of emotional recitation rather than on hierarchical prosodic-phonological structure (e.g., intonational phrases, minor phrases, and explicit phonological boundary phenomena; cf. [21,22]). This perspective is motivated by findings from multiple languages showing that vocal expressions of joy are often associated with higher mean fundamental frequency (pitch), greater intensity, and faster tempo, whereas sadness is typically associated with lower values on these parameters [23,24].

Building on this foundation, Kraxenberger et al. (2018) investigated the role of pitch and tempo variation in spoken poetry on listeners’ perception of joy and sadness [25]. Their results confirmed the presence of emotional prosody in poetic recitation and demonstrated its capacity to shape emotional perception even in listeners lacking semantic comprehension. The study thus offers a prosody-based explanatory pathway for sound–emotion associations in poetry. To date, however, follow-up investigations and replications remain scarce, particularly evidence from tonal languages, in which suprasegmental features simultaneously serve lexically contrastive functions, rendering their role considerably more complex than in the extensively studied Indo-European languages. Although current theorizing holds that pitch contours encoding lexical meaning do not fundamentally interact with those conveying emotion, additional data from artistic language use would nevertheless enrich our understanding of these phenomena.

Against this background, the present study constitutes a further exploration of the role of suprasegmental cues in the communication of emotion through poetry, conducted within the context of Mandarin Chinese—the tonal language with the largest number of speakers.

The present study comprises two interconnected experiments and is methodologically inspired by the research paradigm established by Kraxenberger et al. (2018). The first experiment seeks to verify and quantitatively establish the acoustic-prosodic differences between joyful and sad poetry recitation. The second experiment, conducted through an online questionnaire, investigates the degree to which these emotional acoustic-prosodic features shape listeners’ perception of a poem’s overall emotional valence. Rather than directly comparing the explanatory power of the phoneme-frequency, emotional-rhyme, and emotional-prosody accounts, the present study focuses on the acoustic-prosodic account while controlling segmental distribution and rhyme structure within items.

## Experiment 1

### Materials and methods

#### Participants.

Ten native speakers of Mandarin Chinese (5 male, 5 female; age range 20–32 years, M = 25.0, SD = 3.1) were recruited from university recitation and performance societies. They were asked to rate four joyful and four sad poems and then read them aloud expressively. Prior to the experiment, ethical approval was granted by the Human Research Ethics Committee of Northeastern University. Verbal informed consent was obtained from all participants in accordance with the approved protocol. Recruitment took place from June 8 to 30, 2025, and all participants received financial compensation for their time and involvement.

#### Poems.

Eight highly representative poems (or excerpts; the theme and background of each poem are detailed in S1 File) were selected from a larger pool of 30 classical Chinese poems composed before the 18th century. The initial 30 poems were all canonical works included in China’s compulsory education curriculum and thus widely familiar to the general population. Twenty university students then rated these 30 poems for happiness and sadness on an 11-point scale ranging from –5 (extremely sad) to +5 (extremely joyful). The four most joyful poems and the four saddest poems were selected, with absolute mean valence scores exceeding 3.8 in each direction.

#### Recording and analysis.

All recordings were made in a quiet studio. Poems were presented in randomized order, with the full text and brief historical context embedded in PowerPoint slides. Before reading each poem aloud, participants first rated the poem’s perceived degree of joy or sadness on a 9-point Likert scale ranging from 1 (not at all) to 9 (extremely). They then performed an expressive oral reading of the poem. Recordings were made using a Samson C03 directional condenser microphone at a sampling rate of 44,100 Hz and 16-bit depth.

Prior to acoustic analysis, all audio files were manually annotated at the syllable level. The ProsodyPro Praat script [26] was then used to extract acoustic parameters, including syllable duration, from which global net articulation rate (excluding pauses) was calculated. Pitch values were obtained from manually corrected PitchTier objects to minimize the influence of octave jumps and tracking errors. Additionally, three parameters reflecting voice periodicity were measured: Harmonics-to-Noise Ratio (HNR), jitter, and shimmer.

Linear mixed-effects (LME) models were fitted in *R* (version 4.1.3) using the *lme4* [27] and *lmerTest* [28] packages. Each acoustic parameter was modeled separately as the dependent variable. Separate models were constructed for the joyful and sad poem categories, with subjective rating included as a fixed effect to examine the relationship between acoustic variation and perceptual rating gradients. To account for the ordinal nature of the perceptual ratings, subjective ratings were treated as ordered factors using orthogonal polynomial contrasts, allowing us to test for linear and higher-order changes across rating levels. Initial models included speaker and poem as crossed random intercepts; random-effects structures were simplified when necessary to ensure model convergence and parsimony. Model residuals were visually inspected using Q-Q plots. For pitch, F0 values were transformed to semitones relative to each speaker’s mean F0 before being entered into the model.

### Results

The speakers’ emotional ratings of the poems aligned with our expectations. Mean ratings for poems pre-classified as joyful or sad both substantially exceeded the midpoint of the 9-point scale (5): joyful poems, *M* = 6.88, *SD* = 1.02; sad poems, *M* = 6.85, *SD* = 1.11. This indicates that the selected texts elicited emotional responses of considerable intensity.

Pitch variation was modeled using linear mixed-effects models to examine the relationship between acoustic fluctuations and perceptual ratings. To ensure model convergence and parsimony, the final models for both emotion conditions included speaker as the sole random intercept. In the positive emotion condition, ANOVA revealed a significant main effect of joy ratings on semitone-transformed pitch [*F*(4, 35.33) = 8.63, *p* <  .001]. Orthogonal polynomial contrasts confirmed a robust linear trend [*β* = 4.35, *SE* = 0.87, *t*(34.16) = 5.01, *p* <  .001], indicating that pitch rose linearly with higher joy ratings; no higher-order trends reached significance (all *p*s >  .60). In contrast, in the negative emotion condition, ANOVA revealed a significant main effect of sadness ratings on semitone-transformed pitch [*F*(4, 33.29) = 2.87, *p* =  .038]. Orthogonal polynomial contrasts confirmed a significant linear decrease [*β* = -2.65, *SE* = 1.02, *t*(37.06) = -2.59, *p* =  .014], showing that pitch dropped linearly as sadness ratings intensified. No significant quadratic, cubic, or quartic trends were observed (all *p*s >  .30).

Similar models were employed to analyze the relationship between articulation rate (syllables per second, excluding pauses) and perceptual ratings. Likelihood ratio tests indicated that adding poem as a random intercept did not significantly improve model fit for either condition (both *p*s >  .30); thus, to ensure model parsimony and convergence, the final models retained speaker as the sole random intercept. In the positive emotion condition, articulation rate showed a significant increase with joy ratings. Orthogonal polynomial contrasts revealed a significant linear trend [*β* = 0.47, SE = 0.17, *t*(31.48) = 2.81, *p* =  .008], whereas higher-order quadratic, cubic, and quartic trends were non-significant (all *p*s >  .35). In the negative emotion condition, the omnibus Type III ANOVA for the main effect of sadness ratings did not reach the traditional significance threshold [*F*(4, 35.67) = 1.87, *p* =  .138]. Although the omnibus effect was not significant, the planned linear contrast indicated a significant linear decrease [*β* = -0.50, *SE* = 0.22, *t*(38.97) = -2.29, *p* =  .028], suggesting that higher sadness ratings were associated with slower articulation rate. No significant higher-order trends were observed (all *p*s >  .17).

Mean intensity (dB) was modeled with speaker as a random intercept. In the positive emotion condition, Type III ANOVA indicated a significant main effect of joy ratings [*F*(4, 32.74) = 7.73, *p* <  .001]. Orthogonal polynomial contrasts confirmed a robust linear trend [*β* = 8.80, *SE* = 1.70, *t*(31.98) = 5.19, *p* <  .001], indicating that vocal intensity increased linearly with higher joy ratings. Higher-order quadratic, cubic, and quartic trends were non-significant (all *p*s >  .55). In the negative emotion condition, ANOVA revealed a significant main effect of sadness ratings [*F*(4, 33.46) = 3.10, *p* =  .028]. Orthogonal polynomial contrasts confirmed a significant linear decrease [*β* = -9.45, *SE* = 2.84, *t*(37.54) = -3.33, *p* =  .002], showing that vocal intensity dropped linearly as sadness ratings intensified. No higher-order trends reached significance (all *p*s >  .57).

Regarding HNR, LME analyses revealed distinct modulation patterns: the positive emotion condition (retaining both speaker and poem as random intercepts) yielded a significant omnibus main effect of joy ratings [*F*(4, 27.57) = 2.98, *p* =  .036], driven by a significant cubic trend [*β* = 0.80, *SE* = 0.30, *t*(27.09) = 2.70, *p* =  .012]. In contrast, although the omnibus effect was not significant [*F*(4, 31.33) = 2.06, *p* =  .111], the planned linear contrast indicated a significant linear decrease [*β* = -1.91, *SE* = 0.73, *t*(32.99) = -2.64, *p* =  .013], suggesting reduced harmonicity as perceived sadness intensified. This emotional divergence extended to vocal perturbation metrics. While jitter and shimmer remained statistically stable across varying joy intensities (all omnibus *p*s >  .19), sadness-related increases were most evident for jitter, with weaker evidence for shimmer. Specifically, higher sadness ratings linearly predicted greater cycle-to-cycle frequency perturbations in jitter [*β* = 0.010, *SE* = 0.003, *t*(34.94) = 2.92, *p* =  .006; omnibus *F*(4, 32.11) = 3.06, *p* =  .030]. For shimmer, a planned orthogonal polynomial contrast indicated a significant linear increase in amplitude perturbation, although the omnibus effect was not significant [*β* = 0.022, *SE* = 0.011, *t*(33.64) = 2.04, *p* =  .049; omnibus: *F*(4, 31.64) = 1.17, *p* =  .342].

In summary, these results suggest a systematic relationship between emotional perception and vocal production: higher perceived joyfulness was characterized by linear increases in global prosodic cues, including pitch, intensity, and articulation rate. In contrast, greater perceived sadness was associated with lower pitch and intensity, together with a planned linear decrease in articulation rate. Sadness ratings were also linked to changes in voice quality, including reduced harmonicity and increased jitter, with weaker evidence for increased shimmer.

## Experiment 2

The results of Experiment 1 provide foundational insights into the emotional expression of poetry, demonstrating systematic prosodic differences between expressions of joy and sadness. The present experiment extends this investigation by examining how prosodic features contribute to the perception of emotion in poetry while holding each poem’s textual content constant across prosodic conditions, as well as how prosody interacts with semantic content. To this end, we compared native Mandarin listeners with Mandarin-naive Japanese listeners.

The experiment featured three conditions: prosody–semantics congruent, prosody–semantics incongruent, and neutral. In the congruent condition, the emotional prosody of the recitation was consistent with the affective valence conveyed by the poem’s semantic content. In the incongruent condition, prosody was placed in direct conflict with semantics: poems pre-classified as joyful were delivered using sad prosody (lower pitch and slower tempo), whereas poems pre-classified as sad were delivered using joyful prosody (higher pitch and faster tempo). In the neutral condition, expressive prosodic cues were minimized or removed, yielding a largely emotionally neutral rendering.

Participants were divided into two groups based on their linguistic background: native speakers of Mandarin Chinese (the CN group) and native speakers of Japanese with minimal or no Mandarin proficiency (the non-CN group). Because the non-CN participants had minimal access to Mandarin lexical-semantic content, this group allowed us to examine how available acoustic-prosodic cues contribute to emotional judgments when lexical-semantic access is limited.

If the emotional-prosody hypothesis in poetry is valid, the following predictions should be borne out:

(a) For CN participants, who had full access to the poem’s semantic content, the highest emotional valence ratings consistent with the pre-classified emotions were expected to occur in the prosody–semantics congruent condition. In both the incongruent and neutral conditions, ratings were expected to be significantly attenuated and potentially comparable to one another; nevertheless, they may still preserve an overall valence direction congruent with the poem’s pre-classified emotion, given that semantic information is likely to dominate or override conflicting or absent prosodic cues.
(b) For non-CN participants, who had minimal access to Mandarin lexical-semantic content, available prosodic cues were expected to exert the dominant influence on emotional judgment. Their ratings were therefore predicted to track the emotional valence conveyed by prosody to a greater extent than by poem-specific semantic content, irrespective of semantic–prosodic congruence. In the neutral condition, where prosodic cues are minimal or ambiguous, they were expected to show no systematic emotional bias, yielding a pattern of responses markedly different from that of the CN group.

### Materials and methods

#### Stimuli.

A professional female reciter with formal recitation training was invited to re-record all eight poems. Unlike Kraxenberger et al. (2018), who recorded neutral readings and then synthetically manipulated F0 and tempo to create joyful and sad versions, we instructed the speaker to read each poem directly in three distinct expressive styles: joyful, sad, and neutral. This yielded the required stimuli for the experiment.

This approach was adopted for two main reasons. First, the present study aimed to examine the contribution of expressive acoustic-prosodic realization while holding segmental distribution and rhyme patterns constant. Because the same eight poems were used across prosodic conditions, segmental and rhyme features were controlled within items, whereas the audible prosodic realization was systematically varied. A descriptive analysis indicated that segmental composition did not bias the comparison between emotional groups (see S2 File). Second, pilot attempts to synthetically modify pitch and tempo noticeably reduced the naturalness of the audio. We therefore opted for authentic human performance rather than acoustic manipulation, a strategy made feasible by the speaker’s professional training.

We further conducted supplementary tests to validate the perceptual effectiveness and acoustic benchmarks of the neutral condition. An independent survey was developed to evaluate the emotional state and natural fluency of the neutral vocal stimuli on a 100-point scale (ranging from 0 to 100). This survey consisted of two versions, each incorporating vocal stimuli from the other two experimental conditions as filler items to counteract response bias and adaptation effects. Eleven graduate students majoring in Broadcasting and Hosting were recruited as expert raters (one was excluded due to incomplete data, yielding a final panel of ten). The choice of professional announcers as raters leverages their systematic training in neutral delivery, ensuring a superior capacity to evaluate emotional tendencies based strictly on prosodic features.

Descriptive statistics confirmed that the neutral condition scored substantially lower in emotional richness (M = 22.2, SD = 10.2) than both the congruent (M = 74.2, SD = 16.2) and incongruent conditions (M = 73.4, SD = 17.9), while natural fluency remained uniformly high and comparable across all three conditions (neutral: M = 84.0, SD = 19.8; congruent: M = 85.0, SD = 13.9; incongruent: M = 79.2, SD = 20.0). To statistically validate these differences, a two-way ANOVA on emotional richness ratings, with poem and condition as factors, was conducted, revealing a significant main effect of condition, *F*(2, 136) = 282.76, *p* <  .001, and no significant poem × condition interaction, *F*(14, 136) = 0.81, *p* =  .660. Tukey-adjusted post hoc comparisons further confirmed that the neutral condition was rated significantly lower than both the congruent and incongruent conditions, *p*s <  .001, whereas the congruent and incongruent conditions did not differ significantly, *p* =  .968. The same analysis on natural fluency ratings showed no significant main effect of condition, *F*(2, 136) = 1.03, *p* =  .361, indicating that natural fluency was comparable across conditions. Furthermore, the inter-rater reliability analysis demonstrated good inter-rater reliability among the judges for emotional richness, as indicated by single-measure ICC(2,1) = 0.78, 95% CI [0.64, 0.89]. The expert raters were blind to the condition labels and to the hypotheses of the study, and the audio stimuli were presented in randomized order. The rating results (see S1 Fig) indicated that the neutral audio stimuli were generally perceived as lacking emotional color and were statistically distinguishable from the two emotional conditions.

To establish acoustic benchmarks, we recorded additional neutral speech samples from eight female speakers, including the provider of the stimuli used in this experiment (see S1 Table). While the provider’s mean pitch (approximately 190 Hz) was at the lower end of the range observed among the eight female speakers, though still within the normal female speaking range. Instead, her comparatively narrow pitch range reflects a stable, controlled neutral state. In summary, the audio provided by this speaker fulfilled the fundamental requirements of the current study.

According to the measurements, the neutral stimuli had a mean F0 of approximately 190 Hz, a coefficient of variation (CV) for F0 of 0.17, and an average syllable duration of 0.33 seconds. Using these as a reference, the differences between joyful and sad stimuli and neutral stimuli across multiple parameters are presented in Table 1 as factors (e.g., a factor of 1.51 indicates 1.51 times the neutral value). The results reveal the typical prosodic feature contrasts for each type of auditory stimulus, indicating that the speaker successfully rendered the intended prosodic characteristics.

**Table 1:** Comparison of main prosodic measurements for three types of stimuli.

Condition	F0 Mean	F0 CV	Duration Mean
neutral	1	1	1
joyful	1.51	1.59	0.89
sad	0.85	0.76	1.32

Values for joyful and sad speech are expressed as factors relative to neutral speech.

#### Experimental design.

The experiment required both the CN group and the non-CN group to rate all audio stimuli. To prevent practice effects from repeated exposure to the same texts among Chinese participants—who were familiar with the poems—we employed a between-subjects design for the CN group. Three separate Chinese-language questionnaires were created, each containing all eight poems. The audio for each poem corresponded to only one condition (semantically congruent, semantically incongruent, or neutral, with poems pseudo-randomly assigned), ensuring that the poems did not repeat. A Japanese version of the questionnaire was also prepared, using identical audio stimuli but with instructions in Japanese. Due to anticipated constraints on recruiting Japanese participants, a within-subjects design was used for this group: all stimuli were presented in randomized order within a single questionnaire. These increased task demands were offset by higher compensation, which helped maintain participant engagement. Moreover, because the non-CN participants had minimal or no knowledge of Mandarin, their attention was expected to be directed primarily toward acoustic-prosodic rather than lexical content, thereby reducing the likelihood that repeated exposure to the same texts would substantially influence their ratings.

#### Online questionnaire.

Data were collected via an online questionnaire. The introductory page contained the following information:

1. Participation is voluntary, and appropriate remuneration will be provided upon completion.
2. The task must be completed while wearing headphones or in a quiet environment, alone.
3. Listeners should rate the emotional valence conveyed by each poem only after hearing the entire recording.
4. Only anonymous demographic information is collected; there is no privacy risk. Participants may withdraw at any time without data being recorded.
5. Proceeding constitutes informed consent.

After the introduction, participants provided gender, age, dialect background, and their level of Mandarin proficiency (three options: fluent communication possible, know a few words, completely unable to understand).

Each subsequent page presented one audio stimulus in randomized order, accompanied by the instruction: “Please listen attentively to the recited poem and then rate the perceived sentiment orientation on the scale below: –5 = extremely sad, 5 = extremely joyful, 0 = neutral (no clear leaning).” (Japanese version: 「朗読された詩を注意深く聴き、そこから感じ取れる感情の傾向(感情価)を、以下の尺度で評価してください。–5 = 非常に悲しい、5 = 非常に喜ばしい、0 = 中立(どちらの傾向も感じられない)。」)

#### Participants and sample size.

CN participants were recruited on campus, where conscientious completion of the questionnaire was credited toward fulfillment of one course assignment. All had completed China’s compulsory education curriculum and were thus intimately familiar with both the content and emotional tone of the selected classical poems. More than 360 students initially responded (with a quota of 120 per questionnaire version). Following stringent quality control, 309 valid responses were retained (105 + 104 + 100). Exclusion criteria comprised non-native Mandarin speakers (ethnic minorities), completion times outside the 5-12 minutes range (suggesting inattention), and invariant response patterns across items (indicative of random or careless answering). The between-subjects design guaranteed a minimum of 100 high-quality datasets per experimental condition.

Participants for the non-CN group were recruited via the *Crowdworks* platform, which offers efficient matching, anonymity, and convenient payment. A total of 65 complete questionnaires were collected. Of these participants, 52 were male and 13 were female (age: M = 33.5 ± 4.8 years). Five participants reported knowing only a few basic Mandarin greeting words, which were unrelated to the lexical-semantic content of the classical Chinese poems used in the experiment; the remaining participants reported no understanding of Mandarin whatsoever. As a robustness check, we repeated the main analysis after excluding these five participants, and the overall statistical pattern remained unchanged.

#### Statistical analyses.

Statistical analyses were performed in *R* using cumulative link mixed models implemented in the *ordinal* package [29,30], with participants’ emotional ratings treated as an ordinal dependent variable. The fixed-effects structure included *Condition* (congruent / incongruent / neutral), pre-classified *Emotion* (joyful / sad), *Group* (Chinese / Japanese), and the full three-way interaction among these factors. Random intercepts were specified for poem (to account for inherent differences across individual texts) and participant (to control for individual variation in scale use). Models employed the logit link function to estimate cumulative probabilities across rating categories. Where appropriate, significant interactions were followed up with estimated marginal means and pairwise comparisons using the *emmeans* package [31], with Tukey adjustment for multiple comparisons.

### Results

An initial analysis of inter-rater reliability was conducted to ensure the consistency of emotional valence judgments across cohorts. Results indicated that all Kendall’s *W* coefficients were highly significant (*p* <  .001) and ranged from moderate to very strong (0.399 ≤ *W* ≤ 0.770), confirming robust inter-rater reliability across all experimental conditions for both groups.

**Fig 1:**
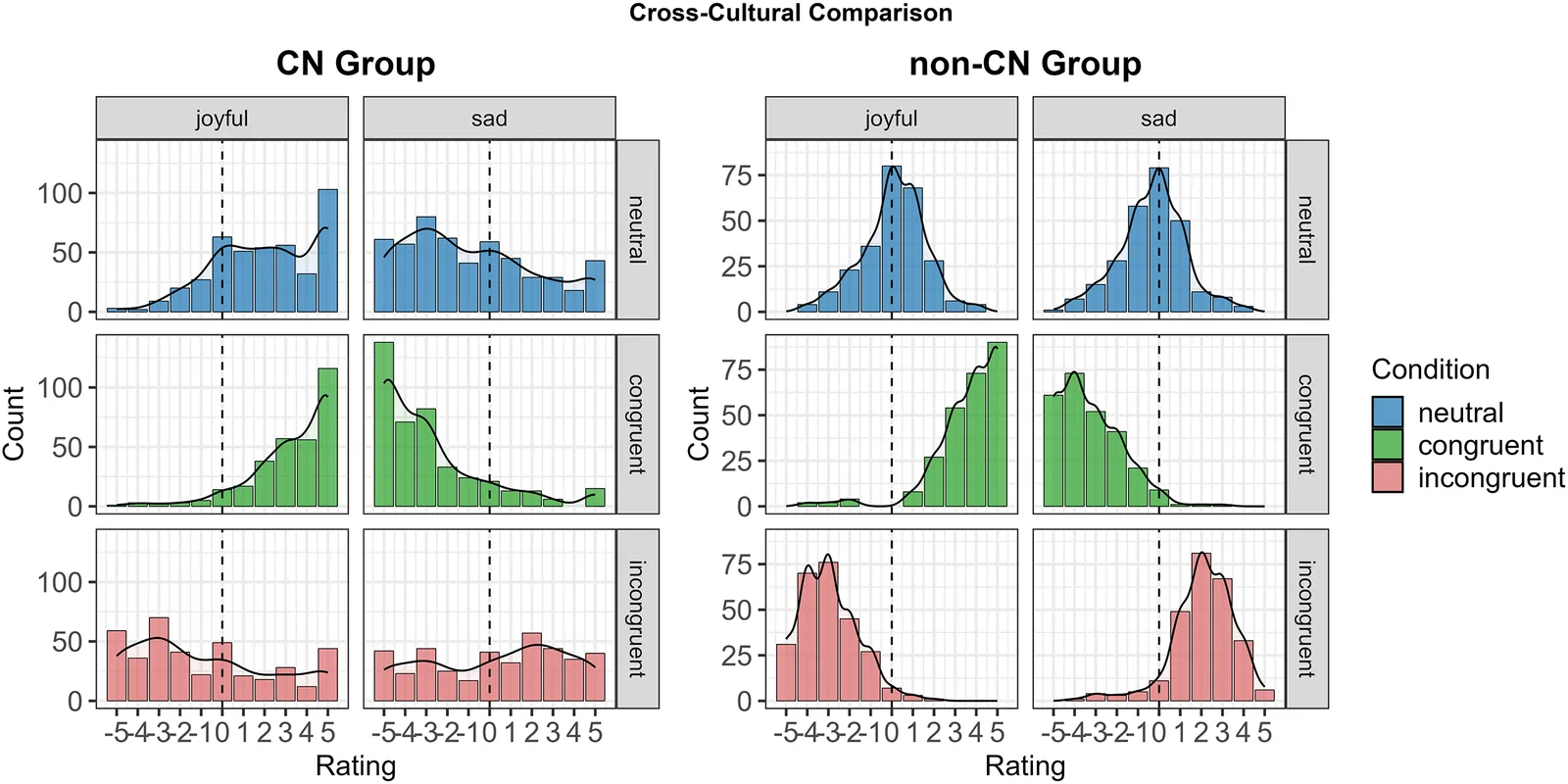
Comparison of rating distributions by emotion and experimental condition in CN and non-CN groups. Bar plots show rating frequency counts (y-axis) from -5 to +5 (x-axis), with conditions color-coded (neutral: blue; congruent: green; incongruent: red). Overlaid density curves (black) display smoothed distributions.

To visually explore these reliable emotional responses, Fig 1 presents the frequency distributions of emotional valence ratings from the CN and non-CN participant groups, across two types of poetic stimuli (joyful vs. sad) and three experimental conditions (semantically congruent, semantically incongruent, neutral). Ratings were measured on a bipolar scale ranging from -5 (extremely sad) to 5 (extremely joyful), with bar plots and overlaid kernel density curves illustrating the count of responses for each rating value. The left panel displays results for the CN group, while the right panel corresponds to the non-CN group; within each group, columns differentiate between joyful and sad poetic stimuli, and rows represent the three experimental conditions.

#### Rating characteristics of the CN group.

For the CN group, emotion ratings were jointly shaped by both the semantic content of the poems and the prosodic cues of the vocal performance, with distinct distribution patterns across conditions. In the neutral prosody condition, ratings for joyful poems showed an overall positive distributional shift, with response frequency rising from the negative to positive pole of the scale and the modal response concentrated at the extreme positive end (+5). By contrast, ratings for sad poems under neutral prosody showed a clear negative shift, peaking around -4 but extending toward neutral and positive values.

In the prosody–semantic congruent condition, rating distributions showed pronounced polarization consistent with the intended emotional valence of the stimuli: for joyful poems, responses were heavily concentrated in the positive range (0 to +5), with the density curve peaking at +5; for sad poems, responses were strongly clustered in the negative range (-5 to 0), with the peak at -5. This pattern was markedly more prominent than the mild directional shifts recorded in the neutral prosody condition, suggesting that congruent semantic and prosodic cues strongly reinforced consistent emotional perception in native speakers.

In the prosody–semantic incongruent condition, rating distributions were markedly more dispersed than in the neutral and congruent conditions. For both joyful and sad poem stimuli, participants’ ratings were influenced by stimulus prosody but remained modulated by the poems’ semantic valence, producing affective judgments that were partly misaligned with the poems’ semantic emotional content. This prosodic effect was moderate in magnitude and yielded broadly distributed responses across the -5 to +5 valence scale rather than a consistently polarized pattern. This pronounced dispersion reflected perceptual conflict experienced by native speakers when processing competing semantic and prosodic emotional cues.

#### Rating characteristics of the non-CN group.

For the non-CN group, the distribution of emotional valence ratings was consistent with dominant reliance on available acoustic-prosodic cues.

In the neutral prosody condition, ratings for both joyful and sad poems exhibited highly similar, approximately symmetric distributions, with clear peaks centered around the neutral zero point (0). Most responses fell within the narrow range of -2 to +2, with few extreme ratings recorded. This pattern indicates that participants in the non-CN group did not perceive a consistent directional emotional valence from stimuli presented with neutral prosody.

In the semantically congruent condition, rating distributions closely followed the emotional direction of the prosodic cues. For joyful prosody paired with joyful semantic content, responses were strongly concentrated toward the positive end of the scale, with the density curve peaking around +4 to +5 and most values falling in the positive range. For sad prosody paired with sad semantic content, responses were strongly clustered toward the negative end, with the density curve peaking around -3 to -4 and the vast majority of values falling in the negative range. Critically, a similar prosody-aligned pattern was observed in the semantically incongruent condition. When joyful semantic content was paired with sad prosody, ratings were strongly concentrated toward the negative end of the scale, peaking in the negative range and aligning with the sad prosody. Conversely, when sad semantic content was paired with joyful prosody, ratings clustered strongly toward the positive end. Compared with the CN group, the non-CN group showed less evidence of semantic–prosodic conflict in this condition. This pattern is consistent with the interpretation that their emotional judgments were primarily guided by acoustic-prosodic cues.

#### Statistical validation.

A cumulative link mixed model was further employed to confirm the observations described above. Likelihood-ratio testing showed that removing the three-way interaction between Emotion, Condition, and Group significantly worsened model fit, *χ²(2)* = 100.14, *p* <  .001. The full model including the three-way interaction yielded a lower *AIC* (16733.24) and was therefore retained for subsequent analyses.

Post hoc comparisons revealed the following patterns. Across the congruent and incongruent conditions, both participant groups showed significant differences in ratings between poems pre-classified as joyful and sad. In the congruent condition, pre-classified joyful poems received significantly higher (more positive) ratings than pre-classified sad poems (CN: *β* = 5.19, *SE* = 0.27, *z* = 19.19, *p* <  .0001; non-CN: *β* = 5.36, *SE* = 0.28, *z* = 19.36, *p* <  .0001). The opposite pattern emerged in the incongruent condition (CN: *β* = -1.12, *SE* = 0.26, *z* = -4.31, *p* <  .0001; non-CN: *β* = -3.60, *SE* = 0.27, *z* = -13.49, *p* <  .0001). In the neutral condition, the non-CN participants showed no significant difference in ratings between the two emotional categories (*β* = 0.28, *SE* = 0.26, *z* = 1.10, *p* =  .27), whereas the CN group continued to rate pre-classified sad poems significantly lower than joyful ones (*β* = 2.40, *SE* = 0.25, *z* = 9.48, *p* <  .0001).

For pre-classified joyful poems, both groups gave significantly higher ratings in the congruent condition than in either the incongruent condition (CN: *β* = 3.31, *SE* = 0.19, *z* = 17.74, *p* <  .0001; non-CN: *β* = 5.01, *SE* = 0.17, *z* = 30.01, *p* <  .0001) or the neutral condition (CN: *β* = 0.61, *SE* = 0.20, *z* = 3.10, *p* <  .01; non-CN: *β* = 2.81, *SE* = 0.15, *z* = 18.24, *p* <  .0001). Moreover, ratings in the incongruent condition were significantly lower than in the neutral condition for both groups (CN: *β* = -2.70, *SE* = 0.19, *z* = -14.36, *p* <  .0001; non-CN: *β* = -2.20, *SE* = 0.15, *z* = -15.03, *p* <  .0001). In short, pre-classified joyful poems received the highest joyfulness ratings when prosody and semantics were congruent, and the lowest (often perceived as sad) when they were incongruent. Similarly, for pre-classified sad poems, both groups assigned significantly lower (more negative) ratings in the congruent condition than in the incongruent condition (CN: *β* = -3.00, *SE* = 0.19, *z* = -16.23, *p* <  .0001; non-CN: *β* = -3.94, *SE* = 0.16, *z* = -25.17, *p* <  .0001) or the neutral condition (CN: *β* = -2.18, *SE* = 0.18, *z* = -12.08, *p* <  .0001; non-CN: *β* = -2.27, *SE* = 0.15, *z* = -15.29, *p* <  .0001). Ratings in the incongruent condition were significantly higher (more joyful) than in the neutral condition for both groups (CN: *β* = 0.82, *SE* = 0.17, *z* = 4.92, *p* <  .0001; non-CN: *β* = 1.68, *SE* = 0.14, *z* = 11.92, *p* <  .0001). Thus, pre-classified sad poems elicited the strongest sadness ratings when prosody and semantics were congruent, but were more likely to be perceived as joyful when prosody conflicted with semantic content.

Direct cross-group comparisons further revealed distinct emotional appraisal strategies between the two cohorts. Under the neutral condition, the CN group rated joyful poems significantly higher (*β* = 1.79, *SE* = 0.19, *z* = 9.60, *p* <  .0001) and sad poems marginally lower (*β* = -0.33, *SE* = 0.17, *z* = -1.94, *p* =  .053) than their non-CN counterparts. In the congruent condition, both groups achieved a high level of perceptual alignment; however, the non-CN group assigned slightly more positive ratings than the CN group for joyful stimuli (*β* = 0.42, *SE* = 0.20, *z* = 2.12, *p* =  .03), whereas no significant group difference was observed for sad stimuli (*β* = 0.25, *SE* = 0.19, *z* = 1.32, *p* =  .19). The clearest cross-group divergence emerged in the incongruent condition. Compared with the CN group, the non-CN group’s judgments showed stronger alignment with the emotional valence conveyed by vocal prosody: they rated joyful poems paired with sad prosody significantly lower (*β* = 1.29, *SE* = 0.18, *z* = 6.97, *p* <  .0001) and sad poems paired with joyful prosody significantly higher (*β* = -1.19, *SE* = 0.18, *z* = -6.50, *p* <  .0001) than the CN group.

### Discussion

#### Prosody shapes the emotional experience of poetry.

The results of the present study largely align with our predictions and support the contribution of emotional prosody to sound–emotion associations in poetry under controlled textual conditions. Speakers spontaneously modulate pitch and tempo to express basic emotions of joy and sadness, and these acoustic variations reliably influence listeners’ emotional interpretations. In the production experiment (Experiment 1), speakers conveyed positive emotions such as joy and exhilaration by raising pitch and increasing articulation rate or tempo; conversely, they expressed sorrow or grief by lowering pitch and slowing the tempo. In addition, sadness was associated with reduced harmonicity (HNR) and increased cycle-to-cycle frequency perturbation (jitter), suggesting that vocal roughness or breathiness may further distinguish sad from joyful recitation.

Experiment 2 suggested that expressive prosodic cues play an important role in shaping the emotional experience of poetry. For Japanese listeners with minimal or no access to the lexical-semantic content of classical Chinese poetry, their judgments of sentiment orientation were consistent with dominant reliance on available acoustic-prosodic features, suggesting that affective appraisal can proceed when lexical-semantic comprehension is unavailable. This pattern was further supported by the neutral condition, in which the absence of strongly emotion-specific prosodic cues was associated with no systematic emotional bias. In contrast, native Mandarin listeners continued to derive affective meaning from semantics even when prosody was neutralized. Nevertheless, their responses remained highly sensitive to prosodic manipulation—incongruent prosody substantially modulated or at times shifted the emotion induced by semantic content. This pattern suggests that semantic and prosodic cues can jointly shape emotional judgments, and that incongruent prosody may alter the affective interpretation of semantically meaningful poetry. However, because the present task did not measure reaction times, selective attention, or online conflict processing, this finding should be interpreted as a behavioral effect of cue incongruence rather than as evidence for a specific cognitive mechanism.

Previous research has found that although top-down control can significantly modulate the weights assigned to prosodic and semantic processing, both types of information processing remain automated to a certain extent. This is particularly evident when prosody and semantics carry conflicting emotional information, requiring the brain to engage in conflict monitoring and interference suppression [32,33]. Given that our survey instructed participants to listen carefully to the stimuli before making judgments, the processing of prosodic features may have been prioritized to reduce cognitive load. Related findings from semantic–prosodic Stroop experiments using Chinese emotional words also suggest that when selective attention is required for a specific channel, emotional cues from prosody are more salient and accessible than semantic content. Furthermore, under conflict conditions, prosody exerts stronger interference on semantic processing, indicating that participants’ emotional judgments are more susceptible to prosodic influence [34].

A related study utilizing ERP (Event-Related Potential) technology further found that emotional prosody, compared with semantics, elicited larger amplitudes and faster responses across multiple stages: early auditory processing (N100, P200), semantic integration (N400), and late-stage decision-making (LPC). This demonstrates prosodic salience from early to late processing stages [35]. These findings suggest that listeners may be highly sensitive to salient acoustic configurations, and that this sensitivity may extend beyond specific language families. For instance, a study involving native English speakers in an emotion-rating task also noted that prosodic cues can strongly influence emotional ratings, even when semantic information is also available [36]. Thus, the acoustic properties of prosody, such as rhythm and pitch contours, may show relatively broad cross-linguistic associations with emotional perception, reflecting shared sensitivity to expressive vocal realization. From an evolutionary perspective, the vocal expression of emotion is an instinct inherited from shared biological ancestors [37]. As a fundamental evolutionary function for auditory species, acoustic analysis exhibits strong feed-forward characteristics. Prosodic processing relies on the rapid activation of the auditory cortex and the limbic system, whereas semantics requires deep processing through language centers, involving a longer neural pathway [38].

The processing advantage of emotional prosody may also be rooted in a broader neurophysiological foundation. Regarding the lateralization of cortical prosody perception, the primary hypotheses proposed within the literature each, to varying degrees, emphasize the right hemisphere’s dominance in prosodic—and specifically emotional or affective prosodic—processing [39]. Recently, advances in neuroimaging research on vocal emotion perception have further revealed the existence of voice-sensitive regions specialized in processing affect—termed “Emotional Voice Areas” [40,41]. Notably, these areas are not merely sensitive to the physical properties of sound; they specifically respond to the affective salience of those sounds. This suggests that “vocal processing” and “emotional processing” are not discrete, sequential steps in the brain but are functionally integrated at an early stage of auditory perception. Remarkably, even prior to the full maturation of the visual system, human infants demonstrate an extraordinary ability to discern vocal emotions [42–44].

Taken together, extensive neuroscientific findings underscore functional specialization relevant to the perception of vocal affect. Although the behavioral nature of the present study precludes a direct mapping of the observed effects onto precise neural substrates, these complementary neurophysiological and developmental viewpoints nevertheless converge with our behavioral findings and provide a plausible interpretive framework for understanding listeners’ sensitivity to emotional prosody.

#### Acoustic salience and arousal-related asymmetry.

A further noteworthy observation in Experiment 2 is that both Chinese and Japanese participants showed stronger or more extreme ratings for joyful than for sad prosody. Ratings for joyful prosody were consistently higher in absolute terms than those for sad prosody. Given that prior literature has not widely established a general perceptual bias for positive valence in the adult population, this asymmetry may not only be linked to the heightened acoustic salience of high-arousal joy, but may also stem from the acoustic proximity between neutral speech and sad affect, and potentially further originate from the culturally driven norms that prescribe a restrained expression of sadness.

The acoustic correlates of joy—markedly elevated pitch, greater pitch excursion, and faster tempo—are more exaggerated, making them easier to recognize. Recent research supports this by noting that such high-arousal emotions maintain better recognition accuracy [45]. In our data, this salience is evident in the robust linear elevation of semitone-transformed pitch (4.35), which confirms a highly perceptible acoustic expansion as joy ratings increased.

In contrast, the acoustic correlates of sadness involve more constrained reductions in pitch and tempo, meaning limited drops in these parameters may be perceived merely as “depressed sadness.” In our study, this compressed acoustic range was clearly reflected in both prosodic dimensions. On the one hand, the linear decrease in semitone-transformed pitch was relatively modest (-2.65); on the other hand, higher sadness ratings only linearly predicted a slight deceleration in articulation rate (-0.50). This explanation aligns with recent categorical perception findings showing that discrimination difficulty along the neutral-sadness continuum is significantly higher than along the neutral-happiness or happiness-sadness continua [46]. This acoustic ambiguity between neutral and sad speech likely prevented participants from assigning higher ratings to the sad stimuli.

Importantly, the prosodic expression of sad affect may be further shaped by cultural norms governing poetic recitation. Since sadness in natural speech is not uniformly low-arousal, the “sad prosody” captured in the present study may be more precisely interpreted as a culturally stylized and restrained poetic affective style. In the tradition of classical Chinese poetry recitation, expressions of sorrow are often shaped by aesthetic norms that favor restraint and subtlety, commonly encapsulated by the notion of *Ai er bu shang* (哀而不伤, “sorrow without distress”). This artistic convention may encourage a subdued acoustic profile in sad recitations.

#### Limitations and future directions.

The present study provides evidence that acoustic-prosodic cues influence emotional perception across the two listener groups examined here. However, several theoretical and methodological limitations should be considered before extending these findings beyond the current sample and stimulus set.

Methodologically, the observed group-level differences should be interpreted in light of several design-related factors. First, the binary contrast between “joy” and “sadness” may partly confound valence with arousal, meaning that some observed effects may reflect arousal-related acoustic variation rather than valence-specific processing. Second, although the comparison between the Mandarin and Japanese cohorts was designed to contrast listeners with different degrees of lexical-semantic access, procedural differences in testing format, recruitment context, and the within-subjects design used for the Japanese group limit strong causal interpretation. These factors are unlikely to fully account for the overall pattern of results, but they remain important for contextualizing the findings. In addition, because individualized trial sequences were not recorded, potential order effects could not be directly modeled. Finally, the reliance on stimuli produced by a single female reciter limits the generalizability of the results. Future studies should adopt more standardized designs, recruit larger and more balanced cohorts, include a wider range of speakers, and examine tone-specific effects on emotion perception.

Beyond these methodological issues, the cognitive and phonological mechanisms underlying the present findings require further clarification. Although the results show systematic associations between surface acoustic cues and emotion judgments, it remains possible that cross-linguistic emotional perception is mediated by prosodic phonological structures, whereby native-language parsing rules interact with acoustic-emotional mappings. For example, Japanese listeners, whose native language uses a pitch-accent system, may automatically interpret sharp pitch changes as markers of word or phrase boundaries [cf. 44], rather than solely as cues to emotional expression. Thus, the present results may reflect an interaction between direct affective decoding and native phonological parsing. Future research should therefore extend this work to more diverse language families, including non-pitch-accent and stress-accent systems, to better disentangle these underlying mechanisms.

Finally, although variation in phoneme frequency and rhyme structure was minimized, the present design does not allow a direct comparison of the explanatory power of prosodic, phoneme-frequency, and rhyme-based accounts. Because textual and performance-related cues are likely to interact, future work should use a larger and more varied corpus of poems to systematically compare the predictive contributions of text-based features, including phoneme frequency and rhyme structure, with those of vocal delivery.

## Conclusion

This study provides empirical support for the role of acoustic-prosodic vocal realization in shaping emotional perception during oral poetry performance. This interpretation is consistent with previous empirical and neurophysiological evidence on vocal emotion perception. In certain contexts, particularly when lexical-semantic access is limited or when prosodic and semantic cues conflict, prosody may exert a strong influence on listeners’ affective responses to poetry.

## Supporting information

S1 File Poems for experimentation. (DOCX)

S2 File Segmental composition of the eight stimulus poems. This HTML report provides a navigable interface for exploring the segmental composition data of the eight stimulus poems, with sortable tables and visualizations organized by emotional valence (Joyful vs. Sad).
(HTML)

S1 Fig Comparison of emotional perception in neutral vs. non-neutral vocal poems. Note: For “emotional richness”, higher scores indicate greater perceived emotional richness in the audio, irrespective of emotional valence. For “natural fluency”, higher scores reflect better perceived fluency in poetic recitation.
(TIF)

S1 Table Acoustic characteristics of neutral speech samples. (DOCX)
