# Supplementary material for: Beyond semantics: Prosody can shape and modulate listeners’ emotional experience of poetry — A cross-linguistic investigation
Source: PLoS One. 2026 Sep 8;21(9):e0345072. doi: 10.1371/journal.pone.0345072 (PMC13552738; doi:10.1371/journal.pone.0345072)
Supplement: Supplementary file 1 [file pone.0345072.s001.docx]

**S1 File. Poems for Experimentation**

| **#** | **Poem Title** | **Text**  **(Original Chinese with IPA transcription)** | **Theme & Content** | **Valence Score** |
| --- | --- | --- | --- | --- |
| 1 | ***New Year’s Day***  *Song Dynasty,*  *Wang Anshi*  *《元日》・宋 王安石* | 爆竹声中一岁除，  [pau̯⁵¹ ʈʂu³⁵ ʂəŋ⁵⁵ ʈʂuŋ⁵⁵ i⁵⁵ suei̯⁵¹ ʈʂʰu³⁵]  春风送暖入屠苏。  [ʈʂʰuŋ⁵⁵ fəŋ⁵⁵ suŋ⁵¹ nu̯an²¹⁴ ʐu⁵¹ tʰu³⁵ su⁵⁵]  千门万户曈曈日，  [tɕʰi̯ɛn⁵⁵ mən³⁵ u̯an⁵¹ xu⁵¹ tʰuŋ³⁵ tʰuŋ³⁵ ʐʅ⁵¹]  总把新桃换旧符。  [tsuŋ²¹⁴ pa²¹⁴ ɕin⁵⁵ tʰɑu̯³⁵ xu̯an⁵¹ tɕi̯əu̯⁵¹ fu³⁵] | This poem vividly depicts the lively scene of the Spring Festival in the Song Dynasty, expressing the festive atmosphere of renewal. The poet uses the imagery of the New Year to convey his hopes for national rejuvenation after political reform. | 6.8 |
| 2 | ***Upon Hearing the Imperial Troops Recapture Henan and Hebei***  *Tang Dynasty, Du Fu*  *《闻官军收河南河北》・唐 杜甫* | 剑外忽传收蓟北，  [tɕi̯ɛn⁵¹ u̯ai̯⁵¹ xu⁵⁵ ʈʂʰu̯an³⁵ ʂou̯⁵⁵ tɕi⁵¹ pei̯²¹⁴]  初闻涕泪满衣裳。  [ʈʂʰu⁵⁵ u̯ən³⁵ tʰi⁵¹ lei̯⁵¹ man²¹⁴ i⁵⁵ ʂaŋ⁵⁵]  却看妻子愁何在，  [tɕʰy̯e⁵¹ kʰan⁵¹ tɕʰi⁵⁵ tsɿ²¹⁴ ʈʂʰou̯³⁵ xɤ³⁵ tsai̯⁵¹]  漫卷诗书喜欲狂。  [man⁵¹ tɕy̯ɛn⁵¹ ʂʅ⁵⁵ ʂu⁵⁵ ɕi²¹⁴ y⁵¹ kʰu̯ɑŋ³⁵]  白日放歌须纵酒，  [pai̯³⁵ ʐʅ⁵¹ fɑŋ⁵¹ kɤ⁵⁵ ɕy⁵⁵ tsuŋ⁵¹ tɕi̯əu̯²¹⁴]  青春作伴好还乡。  [tɕʰiŋ⁵⁵ ʈʂʰu̯ən⁵⁵ tsu̯o⁵¹ pan⁵¹ xɑu̯²¹⁴ xu̯an³⁵ ɕi̯ɑŋ⁵⁵]  即从巴峡穿巫峡，  [tɕi⁵¹ tsʰuŋ³⁵ pa⁵⁵ ɕi̯a³⁵ ʈʂʰu̯an⁵⁵ u⁵⁵ ɕi̯a³⁵]  便下襄阳向洛阳。  [pi̯ɛn⁵¹ ɕi̯a⁵¹ ɕi̯aŋ⁵⁵ i̯aŋ³⁵ ɕi̯aŋ⁵¹ lu̯o⁵¹ i̯ɑŋ³⁵] | This poem expresses the poet’s overwhelming joy upon hearing that the imperial troops have recaptured the Central Plains after the An Lushan Rebellion. It also reflects a deep yearning for national unity and peace. | 6.6 |
| 3 | ***After Passing the Imperial Exam***  *Tang Dynasty,*  *Meng Jiao*  *《登科后》・唐 孟郊* | 昔日龌龊不足夸，  [ɕi⁵⁵ ʐʅ⁵¹ u̯o⁵¹ ʈʂʰu̯o⁵¹ pu⁵¹ tsu³⁵ kʰu̯a⁵⁵]  今朝放荡思无涯。  [tɕin⁵⁵ ʈʂau̯⁵⁵ faŋ⁵¹ taŋ⁵¹ sʅ⁵⁵ u³⁵ i̯a³⁵]  春风得意马蹄疾，  [ʈʂʰuŋ⁵⁵ fəŋ⁵⁵ tɤ³⁵ i⁵¹ ma²¹⁴ tʰi³⁵ tɕi³⁵]  一日看尽长安花。  [i⁵⁵ ʐʅ⁵¹ kʰan⁵¹ tɕin⁵¹ ʈʂʰɑŋ³⁵ an⁵⁵ xu̯a⁵⁵] | This poem directly expresses the poet’s elation and pride after passing the imperial examination. It captures the release of years of suppressed frustration and has become a poetic symbol of the scholarly ideal of success in the civil service system. It reflects the pursuit of official rank and the emotional release upon achieving it. | 7 |
| 4 | ***Four Joys***  *Ming Dynasty,*  *Wang Zhu*  *《四喜诗》・明 汪洙* | 久旱逢甘雨，  [tɕi̯əu̯²¹⁴ xan⁵¹ fəŋ³⁵ kan⁵⁵ y²¹⁴]  他乡遇故知。  [tʰa⁵⁵ ɕi̯ɑŋ⁵⁵ y⁵¹ ku⁵¹ ʈʂʅ⁵⁵]  洞房花烛夜，  [tuŋ⁵¹ fɑŋ³⁵ xu̯a⁵⁵ ʈʂu³⁵ i̯ɛ⁵¹]  金榜题名时。  [tɕin⁵⁵ pɑŋ²¹⁴ tʰi³⁵ miŋ³⁵ ʂʅ³⁵] | Centered around four great joys in life, this poem uses typical scenarios to encapsulate the traditional Chinese ideal of happiness. It reflects a collective cultural understanding of life’s most fulfilling moments. | 7.1 |
| 5 | ***Spring View***  *Tang Dynasty,*  *Du Fu*  *《春望》・唐 杜甫* | 国破山河在，  [ku̯o³⁵ pʰu̯o⁵¹ ʂan⁵⁵ xɤ³⁵ tsai̯⁵¹]  城春草木深。  [ʈʂʰəŋ³⁵ ʈʂʰu̯ən⁵⁵ tsʰau̯²¹⁴ mu⁵¹ ʂən⁵⁵]  感时花溅泪，  [kan²¹⁴ ʂʅ³⁵ xu̯a⁵⁵ tɕi̯en⁵¹ lei̯⁵¹]  恨别鸟惊心。  [xən⁵¹ pi̯ɛ³⁵ ni̯ɑu̯²¹⁴ tɕiŋ⁵⁵ ɕin⁵⁵]  烽火连三月，  [fəŋ⁵⁵ xu̯o²¹⁴ li̯ɛn³⁵ san⁵⁵ y̯ɛ⁵¹]  家书抵万金。  [tɕi̯a⁵⁵ ʂu⁵⁵ ti²¹⁴ u̯an⁵¹ tɕin⁵⁵]  白头搔更短，  [pai̯³⁵ tʰou̯³⁵ sɑu̯⁵⁵ kəŋ⁵¹ tu̯an²¹⁴]  浑欲不胜簪。  [xu̯ən³⁵ y⁵¹ pu⁵¹ ʂəŋ⁵¹ tsan⁵⁵] | This poem portrays the desolation of Chang’an during wartime, revealing the destruction caused by conflict. It conveys the poet’s deep sorrow for the decline of the nation and his personal anguish over separation from family and his inability to serve his country in old age. | -7.5 |
| 6 | ***Climbing the Heights***  *Tang Dynasty,*  *Du Fu*  *《登高》・唐 杜甫* | 风急天高猿啸哀，  [fəŋ⁵⁵ tɕi³⁵ tʰi̯ɛn⁵⁵ kɑu̯⁵⁵ y̯ɛn³⁵ ɕi̯ɑu̯⁵¹ ai̯⁵⁵]  渚清沙白鸟飞回。  [ʈʂu²¹⁴ tɕʰiŋ⁵⁵ ʂa⁵⁵ pai̯³⁵ ni̯ɑu̯²¹⁴ fei̯⁵⁵ xu̯ei̯³⁵]  无边落木萧萧下，  [u³⁵ pi̯ɛn⁵⁵ lu̯o⁵¹ mu⁵¹ ɕi̯ɑu̯⁵⁵ ɕi̯ɑu̯⁵⁵ ɕi̯a⁵¹]  不尽长江滚滚来。  [pu⁵¹ tɕin⁵¹ ʈʂʰaŋ³⁵ tɕi̯aŋ⁵⁵ ku̯ən²¹⁴ ku̯ən²¹⁴ lai̯³⁵]  万里悲秋常作客，  [u̯an⁵¹ li²¹⁴ pei̯⁵⁵ tɕʰi̯əu̯⁵⁵ ʈʂʰɑŋ³⁵ tsu̯o⁵¹ kʰɤ⁵¹]  百年多病独登台。  [pai̯²¹⁴ ni̯ɛn³⁵ tu̯o⁵⁵ piŋ⁵¹ tu³⁵ təŋ⁵⁵ tʰai̯³⁵]  艰难苦恨繁霜鬓，  [tɕi̯ɛn⁵⁵ nan³⁵ kʰu²¹⁴ xən⁵¹ fan³⁵ ʂu̯ɑŋ⁵⁵ pin⁵¹]  潦倒新停浊酒杯。  [liɑu̯³⁵ tɑu̯²¹⁴ ɕin⁵⁵ tʰiŋ³⁵ ʈʂu̯o³⁵ tɕi̯əu̯²¹⁴ pei̯⁵⁵] | Through the act of climbing, this poem expresses the loneliness and sorrow of a wandering, aging poet. It embeds personal suffering within the broader context of a turbulent era, linking individual fate with the fate of the nation, giving the poem a profound historical resonance. | -6 |
| 7 | ***Song of Burying the Flowers***  *Qing Dynasty,*  *Cao Xueqin*  *《葬花吟》・清 曹雪芹* | 尔今死去奴收葬，  [ɚ²¹⁴ tɕin⁵⁵ sɿ²¹⁴ tɕʰy⁵¹ nu³⁵ ʂou̯⁵⁵ tsɑŋ⁵¹]  未卜奴身何日亡。  [u̯ei̯⁵¹ pu²¹⁴ nu³⁵ ʂən⁵⁵ xɤ³⁵ ʐʅ⁵¹ u̯ɑŋ³⁵]  奴今葬花人笑痴，  [nu³⁵ tɕin⁵⁵ tsɑŋ⁵¹ xu̯a⁵⁵ ʐən³⁵ ɕi̯ɑu̯⁵¹ ʈʂʰʅ⁵⁵]  他年葬奴知是谁。  [tʰa⁵⁵ ni̯ɛn³⁵ tsɑŋ⁵¹ nu³⁵ ʈʂʅ⁵⁵ ʂʅ⁵¹ ʂuei̯³⁵]  试看春残花渐落，  [ʂʅ⁵¹ kʰan⁵¹ ʈʂʰu̯ən⁵⁵ tsʰan³⁵ xu̯a⁵⁵ tɕi̯ɛn⁵¹ lu̯o⁵¹]  便是红颜老死时！  [pi̯ɛn⁵¹ ʂʅ⁵¹ xuŋ³⁵ i̯ɛn³⁵ lɑu̯²¹⁴ sɿ²¹⁴ ʂʅ³⁵]  一朝春尽红颜老，  [i⁵¹ ʈʂɑu̯⁵⁵ ʈʂʰu̯ən⁵⁵ tɕin⁵¹ xuŋ³⁵ i̯ɛn³⁵ lɑu̯²¹⁴]  花落人亡两不知。  [xu̯a⁵⁵ lu̯o⁵¹ ʐən³⁵ u̯aŋ³⁵ li̯ɑŋ²¹⁴ pu⁵¹ ʈʂʅ⁵⁵] | Through the intertwining of natural imagery and philosophical reflection on life, this poem expresses Lin Daiyu’s lament for her own tragic fate. It also reveals the deeper tragedy of women’s lives being undervalued in feudal society. It is considered a pinnacle of elegiac poetry in classical Chinese literature. | -6.9 |
| 8 | ***To My Son***  *Song Dynasty,*  *Lu You*  *《示儿》・宋 陆游* | 死去元知万事空，  [sɿ²¹⁴ tɕʰy⁵¹ y̯ɛn³⁵ ʈʂʅ⁵⁵ u̯an⁵¹ ʂʅ⁵¹ kʰuŋ⁵⁵]  但悲不见九州同。  [tan⁵¹ pei̯⁵⁵ pu⁵¹ tɕi̯ɛn⁵¹ tɕi̯əu̯²¹⁴ ʈʂou̯⁵⁵ tʰuŋ³⁵]  王师北定中原日，  [u̯aŋ³⁵ ʂʅ⁵⁵ pei̯²¹⁴ tiŋ⁵¹ ʈʂuŋ⁵⁵ y̯ɛn³⁵ ʐʅ⁵¹]  家祭无忘告乃翁。  [tɕi̯a⁵⁵ tɕi⁵¹ u³⁵ u̯ɑŋ⁵¹ kɑu̯⁵¹ nai̯²¹⁴ u̯əŋ⁵⁵] | This poem places personal death in the context of national reunification, highlighting the poet’s deep concern for the fate of the country. It binds family ritual with national destiny, reflecting unfulfilled aspirations. The sorrow of not living to see the nation reunited is transformed into hope for future generations, revealing steadfast belief in the nation’s future despite personal despair. | -7 |

*Note: The valence scores column reports mean valence scores. Negative values indicate perceived sadness, and positive values indicate perceived joy. All eight stimulus poems are also transcribed character by character into the International Phonetic Alphabet (IPA) in the Text column (Standard Mandarin citation-form pronunciations; tone markers excluded from segment counts)*
