# Supplementary material for: Beyond semantics: Prosody can shape and modulate listeners’ emotional experience of poetry — A cross-linguistic investigation
Source: PLoS One. 2026 Sep 8;21(9):e0345072. doi: 10.1371/journal.pone.0345072 (PMC13552738; doi:10.1371/journal.pone.0345072)
Supplement: Supplementary file 2 [file pone.0345072.s004.docx]

**S1 Table. Acoustic Characteristics of Neutral Speech Samples**

| ***V***ariable | *F0* (Hz) | *intensity* (dB) | *articulation rate* (syl/sec) |
| --- | --- | --- | --- |
| ***MEAN*** | 213 | 68.4 | 3.37 |
| ***SD*** | 49.3 | 4 | 0.73 |

*Note: Acoustic benchmarks were modeled on neutral speech produced by eight female speakers.*
