# Supplementary material for: Beyond semantics: Prosody can shape and modulate listeners’ emotional experience of poetry — A cross-linguistic investigation
Source: PLoS One. 2026 Sep 8;21(9):e0345072. doi: 10.1371/journal.pone.0345072 (PMC13552738; doi:10.1371/journal.pone.0345072)
Supplement: Supplementary file 3 [file pone.0345072.s002.html]

Segmental Composition of the Eight Stimulus Poems — Summary Report (Joyful vs. Sad)


# Segmental Composition of the Eight Stimulus Poems — Summary Report

Three-Level Segment Frequency Analysis, Joyful Group vs. Sad Group

Eight stimulus poems were transcribed character-by-character into IPA (tone markers excluded). Frequencies are reported at three levels:
**Level 1** — consonants vs. vowels. Non-syllabic glides /u̯/, /i̯/, /y̯/ are grouped with vowels at this level based on their high sonority.
**Level 2** — seven categories: stops, fricatives, affricates, nasals, laterals, **approximants**, vowels.
**Approximants** in this analysis are defined as the non-syllabic glides /u̯/, /i̯/, /y̯/ — the non-syllabic counterparts of high vowels /u/, /i/, /y/. At Level 2 they form a distinct consonant subclass; at Level 1 they are assimilated to vowels.
**Level 3** — phoneme-level segments, merged at the phonemic level (e.g., /u/ = [u] + [u̯], /a/ = [a] + [ɑ], /ə/ = [ə] + [ɤ]).
Joyful group: Poems 1–4 (132 syllables, 353 segments); Sad group: Poems 5–8 (180 syllables, 518 segments).
Hover over any sunburst sector for exact counts and percentages.

## Group-Level Comparison Tables

### Level 1 — Consonants vs. Vowels (Group Summary)

| Category | Joyful count | Joyful % | Sad count | Sad % | Δ (pp) |
| --- | --- | --- | --- | --- | --- |
| Consonants | 169 | 47.9% | 236 | 45.6% | 2.3 |
| Vowels | 184 | 52.1% | 282 | 54.4% | -2.3 |

### Level 1 — Consonants vs. Vowels (with Per-Poem Inventory)

| No. | Poem | Group | Syllables | Segments | Consonants | Vowels |
| --- | --- | --- | --- | --- | --- | --- |
| 1 | New Year's Day | JOYFUL | 28 | 78 | 40 (51.3%) | 38 (48.7%) |
| 2 | Upon Hearing the Imperial Troops Recaptured Henan and Hebei | JOYFUL | 56 | 159 | 72 (45.3%) | 87 (54.7%) |
| 3 | After Passing the Imperial Examination | JOYFUL | 28 | 65 | 31 (47.7%) | 34 (52.3%) |
| 4 | Four Joys | JOYFUL | 20 | 51 | 26 (51.0%) | 25 (49.0%) |
| 5 | Spring View | SAD | 40 | 117 | 56 (47.9%) | 61 (52.1%) |
| 6 | Climbing the Heights | SAD | 56 | 172 | 75 (43.6%) | 97 (56.4%) |
| 7 | Song of Burying the Flowers | SAD | 56 | 153 | 72 (47.1%) | 81 (52.9%) |
| 8 | To My Son | SAD | 28 | 76 | 33 (43.4%) | 43 (56.6%) |

### Level 2 — Seven Segment Categories

| Category | Joyful count | Joyful % | Sad count | Sad % | Δ (pp) |
| --- | --- | --- | --- | --- | --- |
| Stops | 27 | 7.6% | 43 | 8.3% | -0.7 |
| Fricatives | 42 | 11.9% | 49 | 9.5% | +2.4 |
| Affricates | 37 | 10.5% | 44 | 8.5% | +2.0 |
| Nasals | 61 | 17.3% | 89 | 17.2% | +0.1 |
| Laterals | 2 | 0.6% | 11 | 2.1% | -1.5 |
| Approximants | 49 | 13.9% | 96 | 18.5% | -4.6 |
| Vowels | 135 | 38.2% | 186 | 35.9% | +2.3 |

### Level 3 — Phoneme-Level Segments (click headers to sort)

| Phoneme | Joyful count | Joyful % | Sad count | Sad % | Δ (pp) |
| --- | --- | --- | --- | --- | --- |
| /u/ [u u̯] | 53 | 15.0% | 75 | 14.5% | +0.5 |
| /i/ [i i̯] | 42 | 11.9% | 63 | 12.2% | -0.3 |
| /a/ [a ɑ] | 46 | 13.0% | 57 | 11.0% | +2.0 |
| /n/ | 26 | 7.4% | 58 | 11.2% | -3.8 |
| /ŋ/ | 30 | 8.5% | 29 | 5.6% | +2.9 |
| /ə/ [ə ɤ] | 13 | 3.7% | 25 | 4.8% | -1.1 |
| /e/ [e ɛ] | 9 | 2.5% | 26 | 5.0% | -2.5 |
| /tɕ/ | 11 | 3.1% | 17 | 3.3% | -0.2 |
| /p/ | 9 | 2.5% | 17 | 3.3% | -0.8 |
| /ʅ/ [ʅ ɿ] | 9 | 2.5% | 16 | 3.1% | -0.6 |
| /ʂ/ | 6 | 1.7% | 16 | 3.1% | -1.4 |
| /x/ | 9 | 2.5% | 13 | 2.5% | 0.0 |
| /o/ | 6 | 1.7% | 12 | 2.3% | -0.6 |
| /ɕ/ | 11 | 3.1% | 7 | 1.4% | +1.7 |
| /ʈʂʰ/ | 10 | 2.8% | 7 | 1.4% | +1.4 |
| /tʰ/ | 8 | 2.3% | 6 | 1.2% | +1.1 |
| /ʈʂ/ | 5 | 1.4% | 8 | 1.5% | -0.1 |
| /l/ | 2 | 0.6% | 11 | 2.1% | -1.5 |
| /y/ [y y̯] | 6 | 1.7% | 7 | 1.4% | +0.3 |
| /ts/ | 6 | 1.7% | 6 | 1.2% | +0.5 |
| /t/ | 3 | 0.8% | 8 | 1.5% | -0.7 |
| /f/ | 7 | 2.0% | 4 | 0.8% | +1.2 |
| /k/ | 3 | 0.8% | 7 | 1.4% | -0.6 |
| /ʐ/ | 5 | 1.4% | 4 | 0.8% | +0.6 |
| /s/ | 4 | 1.1% | 5 | 1.0% | +0.1 |
| /tɕʰ/ | 4 | 1.1% | 4 | 0.8% | +0.3 |
| /kʰ/ | 4 | 1.1% | 4 | 0.8% | +0.3 |
| /m/ | 5 | 1.4% | 2 | 0.4% | +1.0 |
| /tsʰ/ | 1 | 0.3% | 2 | 0.4% | -0.1 |
| /pʰ/ | 0 | 0.0% | 1 | 0.2% | -0.2 |
| /ɚ/ | 0 | 0.0% | 1 | 0.2% | -0.2 |

Click any column header to sort by that field (ascending / descending).

Percentages are relative to each group’s total number of segments. Δ = joyful % − sad %, in percentage points. All counts derive exclusively from the author-provided IPA transcriptions. Phonemes are merged at the phonemic level (e.g., /a/ merges [a ɑ], /i/ merges [i i̯]).

**Joyful poems (Poems 1–4)** — three-level segment hierarchy  
Inner ring: consonants vs. vowels  ·  middle ring: seven categories  ·  outer ring: individual segments

**Sad poems (Poems 5–8)** — three-level segment hierarchy  
Inner ring: consonants vs. vowels  ·  middle ring: seven categories  ·  outer ring: individual segments

## Per-Poem Level 1 & Level 2 Summary

| No. | Poem | Group | Syllables | Segments | Consonants | Vowels | Level 2 Breakdown |
| --- | --- | --- | --- | --- | --- | --- | --- |
| 1 | New Year's Day | JOYFUL | 28 | 78 | 40 (51.3%) | 38 (48.7%) | Vowels: 29, Approximants: 9, Nasals: 16, Affricates: 7, Fricatives: 11, Stops: 6 |
| 2 | Upon Hearing the Imperial Troops Recaptured Henan and Hebei | JOYFUL | 56 | 159 | 72 (45.3%) | 87 (54.7%) | Vowels: 58, Approximants: 29, Nasals: 25, Affricates: 18, Fricatives: 18, Stops: 9, Laterals: 2 |
| 3 | After Passing the Imperial Examination | JOYFUL | 28 | 65 | 31 (47.7%) | 34 (52.3%) | Vowels: 28, Approximants: 6, Nasals: 10, Affricates: 8, Fricatives: 7, Stops: 6 |
| 4 | Four Joys | JOYFUL | 20 | 51 | 26 (51.0%) | 25 (49.0%) | Vowels: 20, Approximants: 5, Nasals: 10, Affricates: 4, Fricatives: 6, Stops: 6 |
| 5 | Spring View | SAD | 40 | 117 | 56 (47.9%) | 61 (52.1%) | Vowels: 41, Approximants: 20, Nasals: 21, Affricates: 9, Fricatives: 14, Stops: 10, Laterals: 2 |
| 6 | Climbing the Heights | SAD | 56 | 172 | 75 (43.6%) | 97 (56.4%) | Vowels: 58, Approximants: 39, Nasals: 27, Affricates: 12, Fricatives: 12, Stops: 20, Laterals: 4 |
| 7 | Song of Burying the Flowers | SAD | 56 | 153 | 72 (47.1%) | 81 (52.9%) | Vowels: 57, Approximants: 24, Nasals: 28, Affricates: 15, Fricatives: 19, Stops: 5, Laterals: 5 |
| 8 | To My Son | SAD | 28 | 76 | 33 (43.4%) | 43 (56.6%) | Vowels: 30, Approximants: 13, Nasals: 13, Affricates: 8, Fricatives: 4, Stops: 8 |

All counts derive exclusively from the author-provided IPA transcriptions.

This report was generated with the assistance of Qwen3.8-Max and has been reviewed by the authors.
